# A polycystin-type transient receptor potential (Trp) channel that is activated by ATP

**DOI:** 10.1242/bio.020685

**Published:** 2016-12-23

**Authors:** David Traynor, Robert R. Kay

**Affiliations:** MRC Laboratory of Molecular Biology, Francis Crick Avenue, Cambridge CB1 0QH, UK

**Keywords:** Trp channel, Polycystin-2, Purinergic signalling, ATP, DIF, *Dictyostelium*

## Abstract

ATP and ADP are ancient extra-cellular signalling molecules that in *Dictyostelium* amoebae cause rapid, transient increases in cytosolic calcium due to an influx through the plasma membrane. This response is independent of hetero-trimeric G-proteins, the putative IP3 receptor IplA and all P2X channels. We show, unexpectedly, that it is abolished in mutants of the polycystin-type transient receptor potential channel, TrpP. Responses to the chemoattractants cyclic-AMP and folic acid are unaffected in TrpP mutants. We report that the DIF morphogens, cyclic-di-GMP, GABA, glutamate and adenosine all induce strong cytoplasmic calcium responses, likewise independently of TrpP. Thus, TrpP is dedicated to purinergic signalling. ATP treatment causes cell blebbing within seconds but this does not require TrpP, implicating a separate purinergic receptor. We could detect no effect of ATP on chemotaxis and TrpP mutants grow, chemotax and develop almost normally in standard conditions. No gating ligand is known for the human homologue of TrpP, polycystin-2, which causes polycystic kidney disease. Our results now show that TrpP mediates purinergic signalling in *Dictyostelium* and is directly or indirectly gated by ATP.

## Abstract (precis)

**Summary:** We show that a Trp channel related to the mammalian polycystin channel, rather than a P2X receptor, is responsible for the purinergic stimulation of cytosolic calcium levels in *Dictyostelium* cells.

## INTRODUCTION

ATP and other purines are ancient signalling molecules used widely in animals as neuro-transmitters and also by protozoa and plants for diverse purposes (Burnstock and Verkhratsky, 2009). The signalling pathway typically consists of release of ATP, its detection by cell surface receptors with consequent signal transduction, and destruction of the signal by ecto-ATPases. Two types of ATP receptor are known in mammalian cells: P2X receptors are gated ion channels, which generally allow calcium into the cell and P2Y receptors are G-protein coupled receptors (GPCRs) (Burnstock, 2007).

Calcium signalling also has ancient origins and it is likely that ancestral single-celled eukaryotes were able to produce Ca^2+^ gradients across their plasma membrane using calcium pumps and transporters, and activate calcium entry into the cytoplasm through regulated channels in the plasma membrane and the membranes of internal vesicular stores of Ca^2+^ ions. Changes in free Ca^2+^ ion concentration could then alter the activity of sensitive proteins and processes in the cytoplasm. Present day microbes use calcium signalling in a wide variety of ways and have recognizable homologues in their genomes to many components of calcium signalling found in mammalian cells (Martinac et al., 2008; Collins and Meyer, 2011; Plattner and Verkhratsky, 2015). These ancient signalling processes can be combined so that ATP causes a cytoplasmic calcium increase.

The social amoeba *Dictyostelium discoideum* grows on bacteria or in liquid media as separate cells (Kessin, 2001). These cells respond to starvation by aggregating together by chemotaxis, to form a multicellular mass and ultimately a stalked fruiting body carrying a mass of spores at its top. In the growth phase the cells are chemotactic to folic acid, which guides them to bacteria, and after starvation they become chemotactic to cyclic AMP (cAMP), which is released periodically from aggregation centres, to which it attracts the amoebae. Both folic acid and cAMP are detected through G-protein coupled receptors (Klein et al., 1988; Pan et al., 2016) and set off a variety of intra-cellular signalling responses, including an influx of Ca^2+^. The coordinated movement and differentiation of amoebae into stalk cells and spores during development is controlled by small molecule signalling, including by cAMP, the polyketides DIF and MPBD (Morris et al., 1987; Saito et al., 2006), cyclic-di-GMP (Chen and Schaap, 2012) and GABA/glutamate (Taniura et al., 2006; Anjard and Loomis, 2006).

*Dictyostelium* cells also respond strongly to extracellular ATP and ADP, which both cause an immediate and transient increase in cytosolic Ca^2+^ due to an influx through the plasma membrane (Ludlow et al., 2008, 2009). *Dictyostelium* cells can also release ATP into the medium in micro-molar concentrations (Sivaramakrishnan and Fountain, 2015) and have an ecto-ATPase activity, which degrades ATP (Parish and Weibel, 1980), suggesting that they have a complete set of purinergic signalling components. However, the receptor responsible for the calcium influx in response to ATP is currently unknown.

The most obvious candidate for this ATP receptor is one or more of the five P2X receptors encoded in the genome, four of which have been shown to be ATP-gated calcium channels in heterologous expression experiments (Fountain et al., 2007; Ludlow et al., 2009; Baines et al., 2013). However, these receptors are largely expressed on the intracellular membranes of the contractile vacuole and have a role in its discharge (Fountain et al., 2007; Ludlow et al., 2009; Sivaramakrishnan and Fountain, 2012; Parkinson et al., 2014). Crucially, a mutant with all five P2X receptors knocked out still retains its calcium response to ATP (Ludlow et al., 2009). The *Dictyostelium* genome only carries a limited set of candidate Ca^2+^ signalling proteins (Eichinger et al., 2005; Wilczynska et al., 2005), which include two transient receptor potential (Trp) channels (Clapham, 2003; Hardie, 2007). The nearest human homologues of these *Dictyostelium* proteins are mucolipin and polycistin-2, which are named after the corresponding genetic diseases (Lima et al., 2012, 2014). There is also a two-pore channel and an IP3-like receptor, IplA (Traynor et al., 2000) and two potential stretch-operated channels: MscS is homologous to the bacterial small conductance mechanosensitive channel (Martinac et al., 2008) and a homologue of the eukaryotic Piezo mechanosensitive channel (Coste et al., 2010).

The role of extra-cellular ATP signalling in the *Dictyostelium* life-cycle is not yet clear. ATP has been reported to affect various processes, including cellular aggregation, possibly by enhancing cyclic AMP signalling (Mato and Konijn, 1975; Perekalin, 1977), and recovery from hypo-osmotic stress (Sivaramakrishnan and Fountain, 2015).

We sought to identify the channel mediating the purinergic response of *Dictyostelium* cells by knocking out candidate calcium channels and assessing the response of the mutant cells to ATP using a reporter for cytoplasmic calcium. In this way we show that the polycystin-type Trp channel, TrpP is essential for the response, and either is the ATP receptor, or closely coupled to it. We also show for the first time that a number of endogenous effector molecules including DIF, GABA and cyclic-di-GMP trigger calcium signals, and that these responses are independent of TrpP.

## RESULTS

### Visualising calcium signalling using the cameleon FRET reporter

**Fig. 1.**
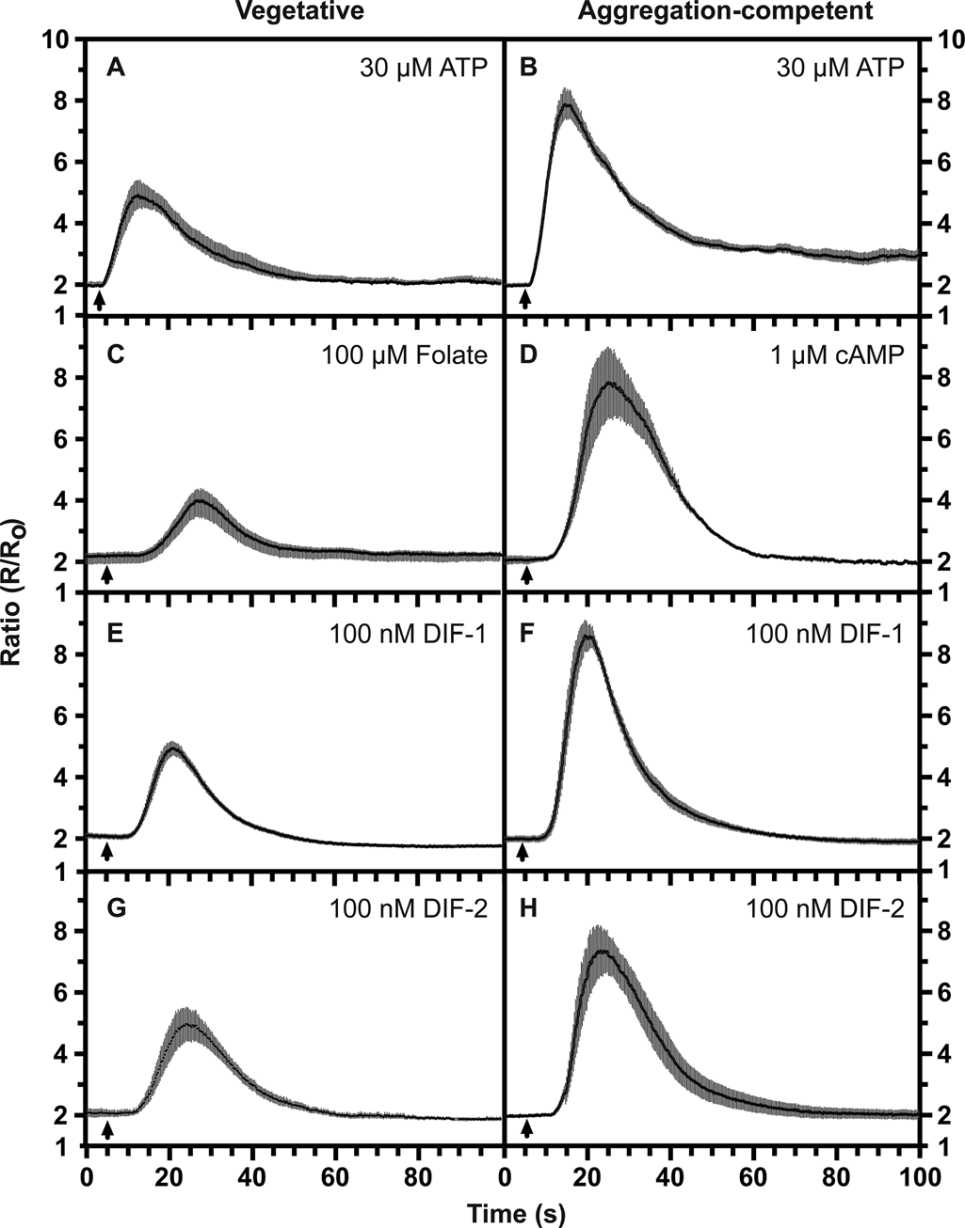
**Ligand-induced calcium signalling in *Dictyostelium* Ax2 cells.** Changes in cytosolic calcium, [Ca^2+^]_c_, in response to different ligands added at the indicated final concentrations: (A,B) 30 µM ATP; (C) 100 µM folate; (D) 1 µM cAMP; (E,F) 100 nM DIF-1; (G,H) 100 nM DIF-2. Vegetative cells (A,C,E,G) or aggregation-competent cells (B,D,F,H) were used. Cells expressing the cameleon YC2.60 FRET reporter for [Ca^2+^]_c_ were stimulated with ligand and the ratiometric changes in fluorescence measured, with each panel showing the mean ratio±s.e.m. (grey bars) of 6-20 cells. The data is representative of at least four independent experiments. The arrow indicates when the compound was added.

In order to characterise the Ca^2+^ signalling triggered by ATP, we first set up a convenient assay to measure changes in cytosolic calcium concentration. The cameleon series of FRET-based, genetically encoded calcium reporters do not require loading into cells, nor do they require an added cofactor, unlike aequorin (Nagai et al., 2004; Horikawa et al., 2010). We initially characterized the well-studied response of starved, developing (aggregation-competent) cells to the chemoattractant cAMP (Abe et al., 1988; Milne and Coukell, 1991; Nebl and Fisher, 1997; Nebl et al., 2002) (Fig. 1D). We found that the higher affinity cameleon YC2.60 (K_d_ 95 nM) was not saturated by a maximal dose of cAMP or ATP and could robustly detect sub-maximal doses, unlike the lower affinity YC3.60 (K_d_ 215 nM) and thus was ideal for our purposes (Fig. S1). Using this reporter, we found that stimulation with a uniform concentration of cAMP causes a transient increase in cytosolic Ca^2+^ levels after a delay of 6.0±1.0 s (mean±s.e.m.; *n*=7), with a peak at 22.1±4.3 s (*n*=7) and return to baseline by 58.1±3.2 s (*n*=7) (Fig. 1D). Half-maximal response is somewhat variable at 87±71 nM (*n*=4) cAMP (Fig. S2).

cAMP signalling is mediated through a family of G-protein coupled receptors, principally cAR1 (Klein et al., 1988). These receptors appear during development and are only expressed at low levels in growing cells, which accordingly show little or no response to cAMP. However vegetative cells do respond to folic acid (Nebl et al., 2002), which is also a chemoattractant and is detected by a G-protein coupled receptor (Pan et al., 2016): as expected, folate induces a delayed Ca^2+^ response, similar to that produced by cAMP (Fig. 1C).

### TrpP mediates the calcium response to ATP

We found that ATP and ADP robustly evoke transient increases in cytosolic Ca^2+^ from both vegetative and aggregation-competent cells (Fig. 1A,B; for a response to ADP, see Fig. 3), confirming previous work (Ludlow et al., 2008, 2009). There is a minimal delay of around 1 s (1.3±0.2 s *n*=7) before onset of the response to ATP, which is similar to the mixing time, and a mean rise time of 11.0±1.4 s (*n*=7) from baseline to peak response. The kinetics of this purinergic response clearly differ from those to folic acid and cAMP, having a much shorter lag before onset. The half-maximal response was at 1.1±0.4 µM (*n*=5) for ATP and 1.6±1.5 µM (*n*=3) for ADP in aggregation-competent cells (Fig. S2). These values were obtained only from those cells producing a response, which was more than 90% at saturating doses, but fell off at lower ATP concentrations (Fig. S2).

**Fig. 2.**
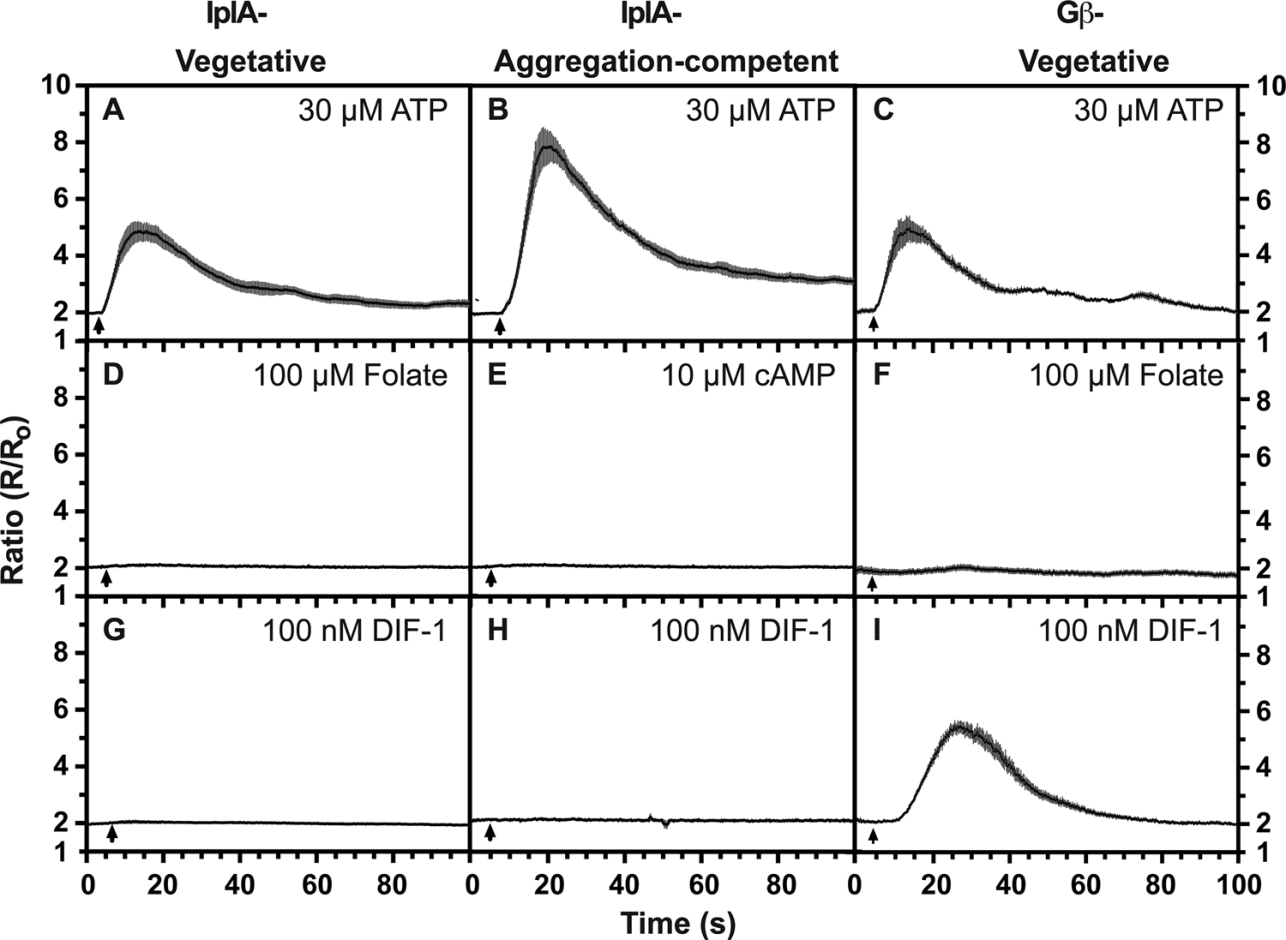
**Requirements for transduction through G_β_ and IplA for calcium signalling.** Mutant cells lacking either G_β_, the only known G_β_ subunit of hetero-trimeric G-proteins in *Dictyostelium*, or IplA, a homologue of the IP3 receptor, were stimulated with the following ligands at the indicated final concentrations: (A-C) 30 µM ATP; (D,F) 100 µM folate; (E) 10 µM cAMP; (G-I) 100 nM DIF-1. Both mutant strains respond to ATP, but the responses to folate and cAMP are abolished (the response of G_β_ null cells to cAMP was not tested). The response to DIF-1 depends on IplA but not G_β_. Vegetative (A,C,D,F,G,I) or aggregation-competent (B,E,H) cells were used. Cells expressing the cameleon YC2.60 FRET reporter for [Ca^2+^]_c_ were stimulated with ligand and the ratiometric changes in fluorescence measured, with each panel showing the mean ratio±s.e.m. (grey bars) of 8-20 cells. The data is representative of at least four independent experiments. The arrow indicates when the compound was added.

Genetically, the response to ATP does not depend on any of the P2X receptors encoded in the *Dictyostelium* genome (Ludlow et al., 2009). To try and establish the signalling route used by ATP we therefore examined the effect of mutating other signalling proteins. First, we tested dependence on hetero-trimeric G-proteins using a null mutant in the only G_β_ subunit encoded in the genome (Wu et al., 1995) and found that the response to ATP was unaffected (Fig. 2C). In contrast the responses to folate and cAMP were abolished (Fig. 2D-F). Similarly, the response in a mutant of IplA, the homologue of the endoplasmic reticulum channel activated by IP3, to ATP was also intact (Fig. 2A,B) (Traynor et al., 2000). Thus, the purinergic response does not appear to require either hetero-trimeric G-proteins or IplA (Ludlow et al., 2008), in both cases differentiating it from the GPCR-mediated responses to cAMP and folate.

**Fig. 3.**
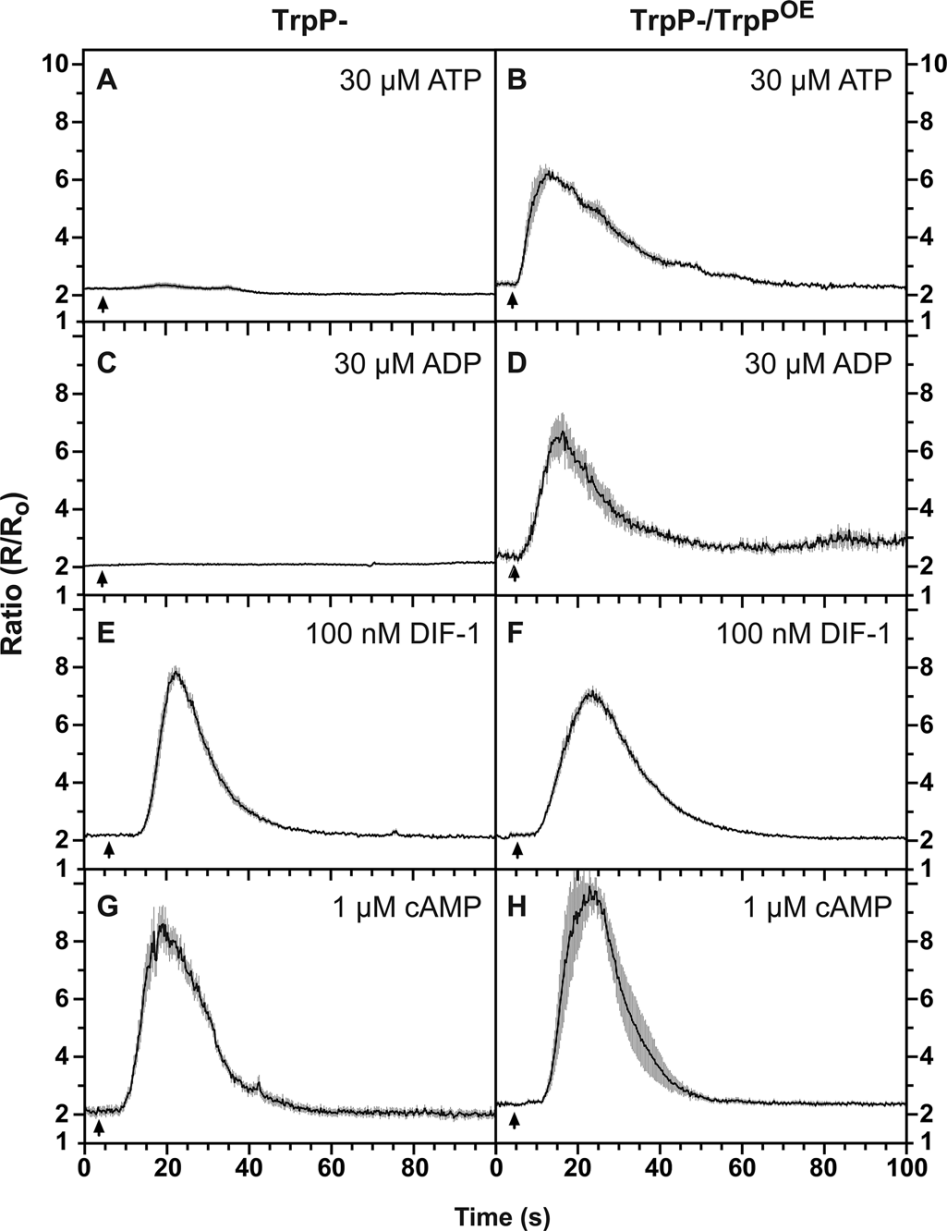
**ATP-stimulated calcium signalling requires the Trp channel, TrpP.** (A,C,E,G) [Ca^2+^]_c_ transients stimulated by ATP (A) and ADP (C) are abolished in TrpP null cells, whereas [Ca^2+^]_c_ transients stimulated by cAMP (G) and DIF-1 (E) are essentially normal. (B,D,F,H) Purinergic responses are restored by over-expression of TrpP in the TrpP- strain (B,D), whereas responses to DIF-1 (F) and cAMP (H) are little affected by over-expression of TrpP. Aggregation-competent TrpP null cells expressing the [Ca^2+^]_c_ reporter cameleon YC2.60 were used, with as indicated, a plasmid for expression of TrpP (pDT50). Changes in the fluorescence ratio with time are presented with each panel showing the mean ratio±s.e.m. (grey bars) of 6-15 cells. The data is representative of at least three independent experiments. The arrow indicates when the compound was added.

We therefore investigated other candidate calcium signalling proteins and made null mutants in three potential channels: the mechano-sensitive channel homologue, MscS (dictyBase DDB_G0277253; http://dictybase.org) and two Trp channels, one a mucolipin homologue, MclN (dictyBase DDB_G0291275) (Lima et al., 2012), the other a polycystin-2 homologue, which we call TrpP (gene, *trpP*; the protein is also known as PKD2; dictyBase DDB_G0272999) (Lima et al., 2014). In case of redundancy, we made a triple mutant lacking all three proteins and in our initial experiments used the uptake of ^45^Ca^2+^ to measure the response. To our surprise, we found that the fast responses to ATP and ADP are essentially abolished in this triple mutant (Fig. S3A). Testing the single mutants individually showed that the rapid ATP and ADP responses are abolished in the TrpP null mutant (Fig. 3A,C; Fig. S3B), but unaffected in the other mutants (Fig. S3C,D).

The abolition of responses to ATP and ADP in *trpP*– mutant cells was confirmed using the cameleon FRET reporter in both vegetative and aggregation-competent cells (Fig. 3A,C; Fig. S4). Responsiveness could be restored by expressing TrpP under the control of its own promoter, demonstrating that the phenotype is due to the loss of TrpP and not a secondary mutation introduced elsewhere in the genome (Fig. 3B,D). In addition, a C-terminal fusion of TrpP to GFP is largely localised to the plasma membrane in living cells, consistent with TrpP acting as a plasma membrane channel (Fig. S5)

Thus we conclude that TrpP mediates the fast calcium responses to ATP and ADP and is likely gated, directly or indirectly, by ATP and ADP.

### Purinergic and chemoattractant Ca^2+^ signalling use genetically distinct pathways

We have shown above that purinergic signalling does not require either G_β_ or IplA, whereas these proteins are required for chemoattractant signalling by folic acid and cAMP – although there are contradictory reports on the importance of G_β_ for cAMP signalling (Milne and Devreotes, 1993; Nebl et al., 2002; Ludlow et al., 2008). Both folate and cAMP calcium signalling depend on an influx of extracellular calcium through the plasma membrane, but the channel responsible has not been identified (Nebl and Fisher, 1997). In principle this channel could be TrpP, to which these ligands might couple indirectly through their respective GPCRs. However, we found that the responses to both cAMP and folate remain intact in TrpP null cells, showing that TrpP is not their influx channel (Fig. 3G; Fig. S4E). Thus the pathways of chemoattractant and purinergic signalling are genetically distinct.

We also noticed that TrpP null cells occasionally show a small, delayed response to ATP, which can also be elicited by buffer alone (Fig. S6; Table S1). A similar delayed response to ATP is seen in wild-type cells treated with Zn^2+^ to inhibit the primary response (Ludlow et al., 2008). This response might be due to a stretch-operated channel, which is activated by the physical stresses of mixing in ligand or buffer. Since the timing of the delayed responses overlaps with those depending on IplA, we created a double *trpP*–/*iplA*– mutant, and found that the response is completely abolished (Fig. S6). Indeed, this double mutant lacks a calcium response to all ligands tested (illustrated for ATP and cAMP in Fig. S6) but remains sensitive to the calmodulin inhibitor, calmidazolium, which is known to elevate cytosolic Ca^2+^ levels by Ca^2+^ release from stores (Schlatterer and Schaloske, 1996).

### Ca^2+^ signalling is induced by DIF, cyclic-di-GMP and GABA independently of TrpP

The differentiation and behaviour of *Dictyostelium* cells during multicellular development is controlled by a number of small signalling molecules, in addition to cAMP, but whether these molecules cause rapid changes in cytosolic Ca^2+^ levels is not known. To determine the scope of calcium signalling mediated by TrpP, we tested whether these endogenous signals could trigger Ca^2+^ signals, and if so, whether the response depends on TrpP, or not.

The DIFs are a family of chlorinated polyketides that induce stalk cell differentiation during development (Morris et al., 1987; Masento et al., 1988), particularly those of the fruiting body basal disc (Saito et al., 2008). DIF has rapid effects on protein phosphorylation and transcription (Sugden et al., 2015; Williams et al., 1987) but its receptor is unknown.

We found that DIF-1, the major species, causes a robust increase in cytosolic Ca^2+^ in aggregation-competent cells after a delay of about 5.5±1.2 (*n*=7) seconds, reaching a peak at around 15.5±2.1 (*n*=7) seconds (Fig. 1F). Half-maximal response is at around 20 nM (Fig. S1; EC_50_ 23.3±3.7 nM; *n*=3), which is well within the range inducing cell differentiation and below the estimated physiological concentration (Kay, 1998). Vegetative cells give similar but weaker responses, consistent with the effects of DIF-1 early in development (Fig. 1E) (Wurster and Kay, 1990; Fukuzawa et al., 2001).

DIF-2 also elicits a response from both vegetative and aggregation-competent cells (Fig. 1G,H). It has 40% of the activity of DIF-1 in a cell differentiation assay but was equipotent in causing a Ca^2+^ response in aggregation-competent cells (24.8±13.7 nM, *n*=3; Fig. S1), whereas the less potent analogue, DIF-3, only gives a weak response at ≥1 µM, as does an analogue lacking the methyl group (des-methyl DIF-1; not shown). The unmodified polyketide (THPH) from which DIF-1 is synthesised (Kay, 1998) produces no response up to 10 µM.

The response to DIF is totally dependent on IplA in both vegetative and aggregation-competent cells but is maintained in G_β_ null cells, suggesting that it does not depend on a heterotrimeric G-protein (Fig. 2G-I). The response also remains essentially unchanged in TrpP null cells and so does not depend on TrpP (Fig. 3E,F).

A second polyketide, MPBD, is important both during early development and for proper spore maturation (Saito et al., 2006; Narita et al., 2014). It failed to evoke a calcium response from vegetative and aggregation-competent cells at 1 µM (not shown).

**Fig. 4.**
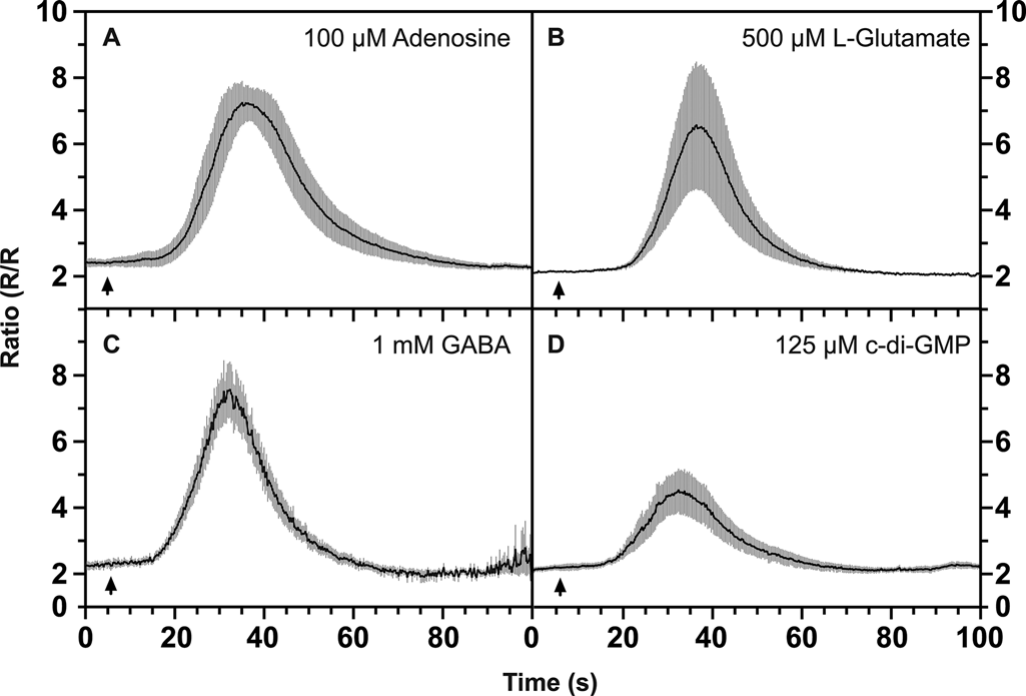
**Calcium responses to adenosine, c-di-GMP, L-glutamate and GABA obtained using aggregation-competent cells.** Changes in cytosolic calcium, [Ca^2+^]_c_, in response to the ligands added at the following final concentrations: (A) 100 µM adenosine; (B) 500 µM L-glutamate; (C) 1 mM GABA; (D) 125 µM c-di-GMP. Cells expressing the cameleon YC2.60 FRET reporter for [Ca^2+^]_c_ were stimulated with ligand and the ratiometric changes in fluorescence measured, with each panel showing the mean ratio±s.e.m. (grey bars) of at least six cells in each of three experiments. The arrow indicates when the compound was added.

We found that cyclic-di-GMP, which is required for stalk cell maturation (Chen and Schaap, 2012; Song et al., 2015), caused a delayed Ca^2+^ response from aggregation-competent cells at 125 µM (Fig. 4D). *Dictyostelium* has a GABA signalling system, which is active during development (Taniura et al., 2006; Anjard and Loomis, 2006; Wu and Janetopoulos, 2013) and we found that GABA and L-glutamate both evoke a cytoplasmic calcium signal from aggregation-competent cells, although this was erratic (Fig. 4B,C). Finally, adenosine, which affects prestalk and prespore patterning in development (Schaap and Wang, 1986) also causes a calcium response (Fig. 4A). The calcium responses to GABA and L-glutamate are independent of TrpP, remaining intact in the null mutant, but do depend on IplA (Fig. S7).

### ATP causes cell blebbing

We sought to establish the role of purinergic signalling in *Dictyostelium* biology by examining, first cellular responses to ATP, and then the TrpP mutant phenotype.

Early reports suggested that extracellular ATP stimulates aggregation centre formation in small drop assays of starving cells (Mato and Konijn, 1975; Perekalin, 1977), but we could not reproduce this effect using up to 1 mM ATP (not shown). We investigated the effects of ATP on cell motility in detail. ATP was not a chemoattractant for aggregation-competent cells at a range of concentrations, and a uniform concentration of ATP did not enhance or inhibit chemotaxis to cAMP (Table S2). Neither was there a significant effect of a uniform concentration of ATP on the speed of chemotaxing cells or on the random movement of either vegetative or starving cells (Table S3).

**Fig. 5.**
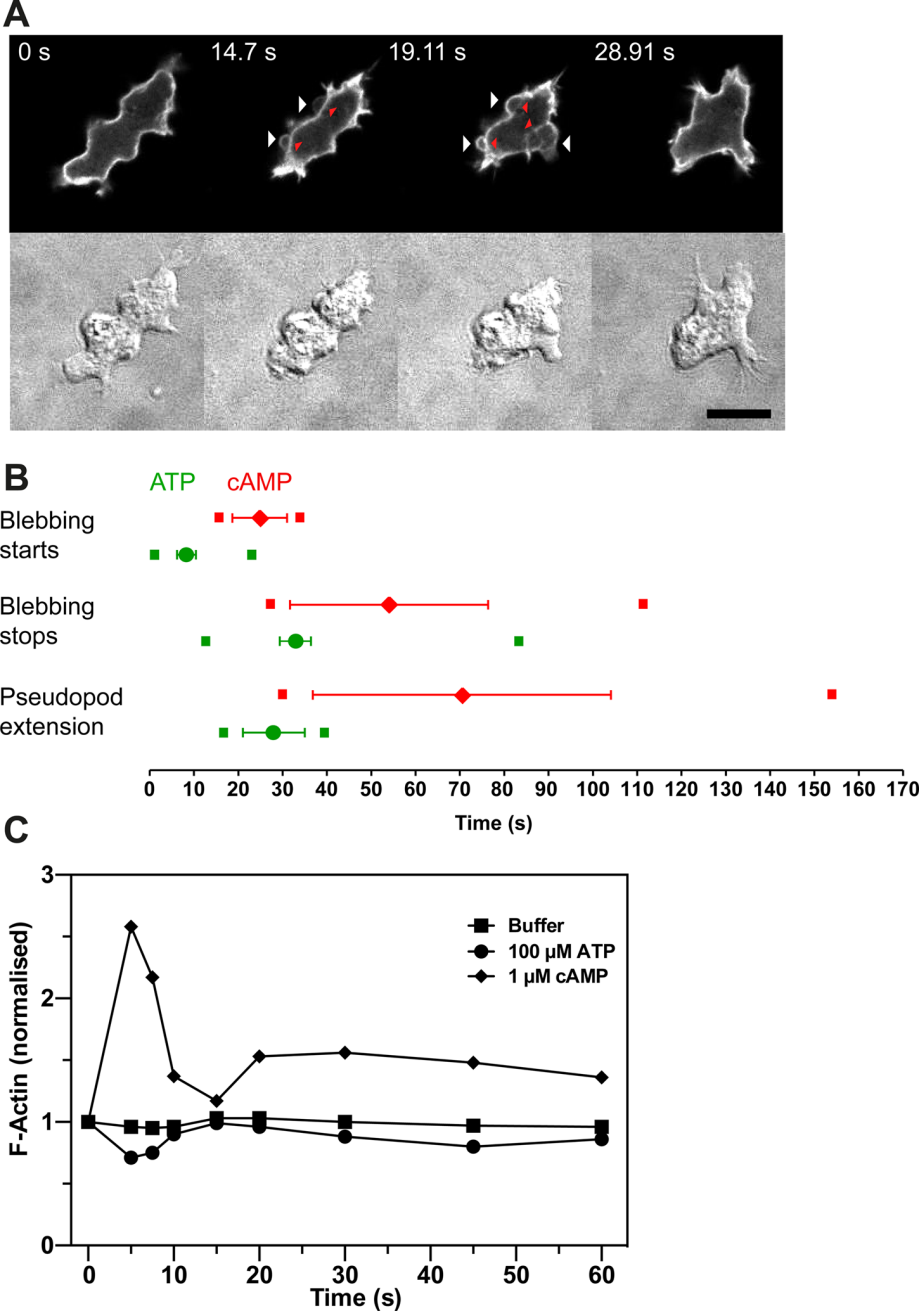
**ATP addition induces cellular blebbing.** (A) Ax2 cell stimulated with 30 µM ATP at t_0_ and expressing the F-actin reporter GFP-ABP120 observed by fluorescence confocal and DIC microscopy. The cell starts to bleb after 14 s (white arrows) and stops by 29 s. When first formed, the blebs are almost devoid of F-actin but leave an F-actin scar behind (red arrowheads). Scale bar: 10 µm. (B) Comparison of the timings of ATP- and cAMP-induced bleb formation (cAMP data reproduced with permission from Langridge and Kay, 2006). The mean±s.e.m. for onset of each of the events are indicated for ATP (green) and cAMP (red). The small squares indicate the times of earliest onset and cessation of each event for the cells (*n*=31) in this data set. (C) Actin polymerization in cells in response to 100 µM ATP, 1 µM cAMP or buffer only. The data shown is representative of three experiments, but the slight F-actin de-polymerization seen in this example did not repeat in response to ATP. Aggregation-competent cells were used throughout.

In the course of the FRET measurements of calcium, we noticed that ATP addition causes cells to bleb vigorously (Fig. 5A). Blebs occur when the plasma membrane becomes detached from the underlying F-actin cortex, and is driven out by fluid pressure. *Dictyostelium* cells can move using blebs instead of pseudopods (Yoshida and Soldati, 2006; Zatulovskiy et al., 2014; Tyson et al., 2014) and blebbing is also induced by cAMP.

Morphologically, blebs induced by ATP resemble those induced by cAMP, with their characteristic rapid expansion, smooth curvature and residual scar of F-actin, representing the former cortex. A new cortex is then rapidly rebuilt on the exposed membrane of the bleb. However, ATP induces blebbing much faster than the published timing for cAMP (Langridge and Kay, 2006), with a delay of only 3-7 s compared to around 25 s for cAMP (Fig. 5B). ATP-induced blebbing requires myosin-II and is abolished in null mutants of either the myosin-II heavy chain or the essential light chain (Fig. S8), as is cAMP-induced blebbing (Zatulovskiy et al., 2014).

The signalling and other events triggered by ATP and cAMP differ significantly: ATP does not cause the transient actin polymerization characteristic of the period before blebbing starts in cells stimulated with cAMP (Langridge and Kay, 2006) (Fig. 5C). Nor does ATP stimulate PIP3 production, as measured in live cells by recruitment of the PH-CRAC reporter to the plasma membrane (Parent et al., 1998), or activate the MAP kinase ErkB (Kosaka and Pears, 1997) or the AKT homologue PKB (Meili et al., 1999), as detected in western blots for the phosphorylated form of the protein kinase (data not shown).

### TrpP mutant phenotype

As an alternative approach to establish the role of purinergic signalling we examined the phenotype of TrpP null cells in detail. A TrpP mutant made by insertional mutagenesis was reported to have a growth defect in HL5 liquid medium (Waheed et al., 2014). We tested the growth of six independent knock-out clones, shaken in HL5 liquid medium: two had modest defects, possibly due to secondary mutations introduced during transformation, but the other four were statistically indistinguishable from wild-type, suggesting that TrpP is not required in any way for axenic growth (Table S2). FITC dextran uptake as a measure of fluid uptake, and phagocytosis of yeast were also indistinguishable from wild-type cells measured in a clone with normal growth (data not shown).

**Fig. 6.**
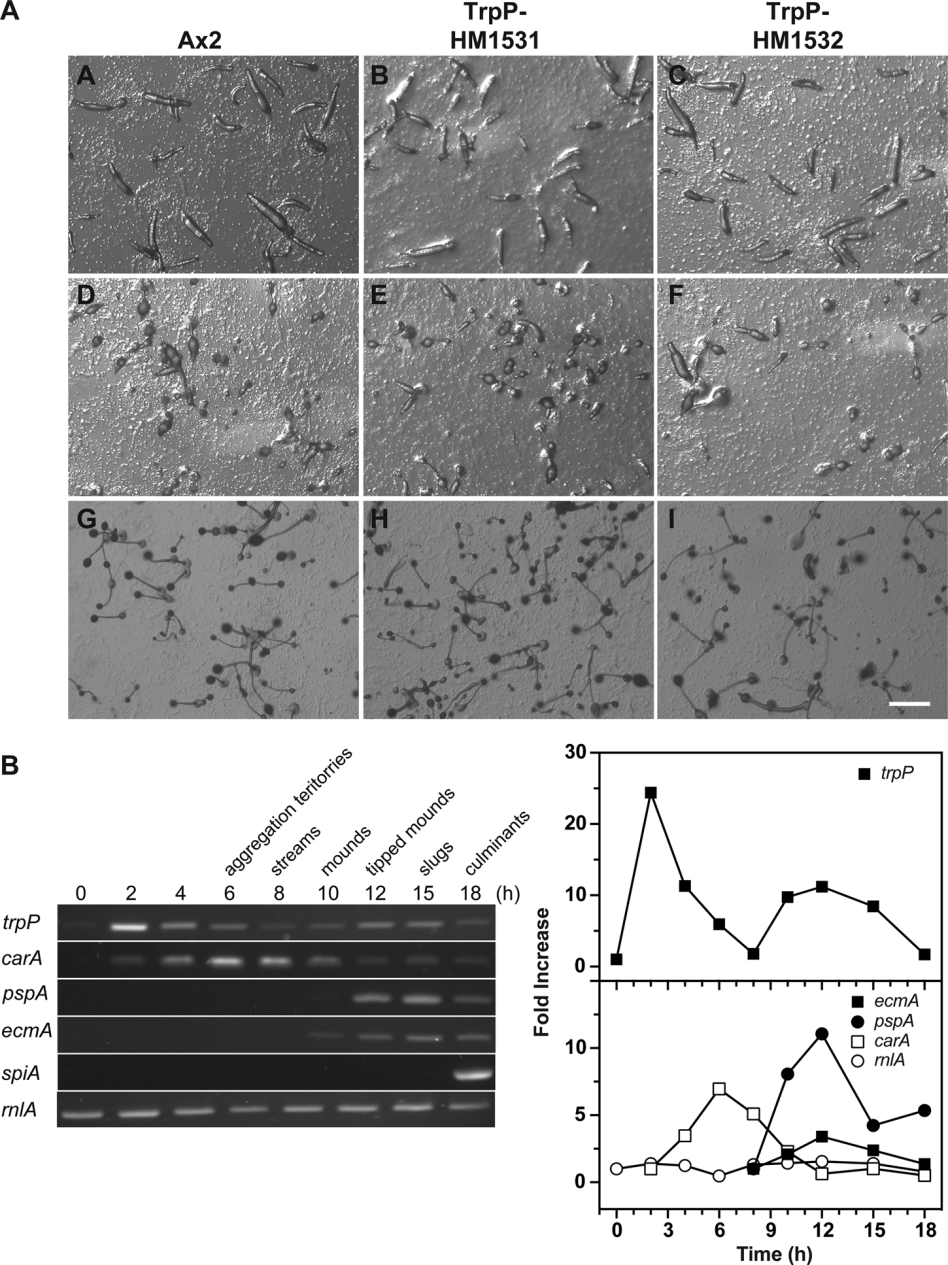
**Development of wild-type and TrpP null cells on agar and developmental expression of TrpP mRNA.** (A) Development of Ax2 parental cells (panels A,D,G); TrpP null cells panels (B,E,H) strain HM1531; and TrpP null cells, strain HM1532 (panels C,F,I). All strains develop with nearly identical timing; the time points shown are: 14 h, slugs; 19 h, early culminants; 23 h, fruiting bodies. Scale bar: 0.5 mm. (B) Developmental regulation of TrpP mRNA in comparison to standard markers as determined by reverse-transcription PCR.

The TrpP gene is expressed at only low levels in growing cells, but the mRNA increases strongly during early development, and then again during later development, suggesting that its main role may lie in development (Fig. 6B) (Parikh et al., 2010). However, overall development of TrpP mutant cells is virtually indistinguishable from wild-type: the timing of different stages and the size of the fruiting bodies are the same, and the only difference we noticed is that the mutant fruiting bodies tended to collapse more frequently (Fig. 6A).

TrpP null cells had normal chemotactic parameters in both steep and shallow cAMP gradients in paired comparisons with wild-type cells (cells were placed at different parts of the same chemotaxis chamber; Tables S2, S3) and moved with only slightly reduced speed and chemotactic efficiency under an agarose overlay, which provides mechanical resistance and causes cells to move using blebs (Table S2) (Zatulovskiy et al., 2014).

Surprisingly, blebbing of TrpP null cells is normal in response to ATP (Fig. S8). This suggests that blebbing is mediated through a further, unidentified ATP receptor.

It has been reported that a TrpP mutant made in the DH1 background is defective in rheotaxis – the movement of cells orientated by liquid flow (Lima et al., 2014). We tested TrpP mutants made in our Ax2 background and found that they are still capable of efficient rheotaxis (Fig. S9).

We conclude that ATP-stimulated Ca^2+^ signalling through TrpP can only have a subtle or redundant effect on growth and development in standard laboratory conditions.

## DISCUSSION

ATP and ADP cause a rapid and transient increase in cytosolic calcium levels in *Dictyostelium* cells (Ludlow et al., 2008, 2009). The major advance described in this paper is the discovery that this response is mediated by the Trp channel TrpP, since in TrpP null mutants the fast calcium response is totally abolished, yet can be restored when the protein is re-expressed. Conversely this purinergic response is independent the G_β_ subunit of heterotrimeric G-proteins and of IplA, the *Dictyostelium* homologue of the IP3-activated calcium release channel.

Although this genetic evidence is clear, direct gating by ATP has not yet been demonstrated by electrophysiology with heterologously expressed TrpP, and so there remains a formal possibility that gating is indirectly mediated by another protein. However, the specific requirement for TrpP in ATP responses, and the lack of effect of TrpP mutation on chemoattractant, DIF, cyclic-di-GMP or GABA signalling argues that, as a minimum, TrpP is likely to be dedicated to purinergic signalling.

The purinergic calcium response is significantly different to the responses evoked by the chemoattractants folic acid and cAMP, which are mediated by GPCRs. It seems that at least two basic modes of calcium signalling can be distinguished in *Dictyostelium*: ‘GPCR-dependent’ signalling (cAMP and folic acid) whose onset is delayed for 5-10 s after the stimulus, and which depend on IplA and at least partially on G_β_; and ‘purinergic’ signalling (ATP and ADP) which has a rapid onset of less than 1 s, is independent of G_β_ and IplA, but depends on TrpP.

We also show for the first time that the stalk cell-inducing morphogen DIF (Morris et al., 1987), whose receptor is unknown, causes a fast, transient calcium response in the physiological concentration range. The characteristics of this response – delayed and IplA-dependent – are more consistent with the G-protein-dependent mode of signalling, but surprisingly we find that this response is largely independent of G_β_. Although not studied in detail we also found delayed calcium responses to di-cyclic-GMP (Chen and Schaap, 2012), L-glutamate and GABA (Anjard and Loomis, 2006) but there was no response to the polyketide MPBD (Saito et al., 2006).

TrpP is well conserved between dictyostelid species (Sucgang et al., 2011; Heidel et al., 2011; Urushihara et al., 2015) arguing that purinergic signally must also have a conserved role. We found that ATP is not a chemoattractant for aggregation-competent cells and does not modulate chemotaxis to cAMP, nor could we detect a chemotactic defect in TrpP null mutants. Growth of TrpP null cells in liquid medium was also normal, contrary to a recent report (Waheed et al., 2014) and development only slightly perturbed. It has been reported that TrpP is required for rheotaxis (Lima et al., 2014) but TrpP mutants made in our laboratory strain showed no such defect. Such discrepancies are not unknown in the *Dictyostelium* literature, and are most likely accounted for by genetic background effects, or secondary mutations introduced during gene knock-out (Bloomfield et al., 2008; Schilde et al., 2004; Pollitt et al., 2006; Sivaramakrishnan and Fountain, 2013).

The one clear effect we can detect of adding ATP or ADP to cells is to induce almost immediate blebbing. Blebs form where the plasma membrane detaches from the underlying cortex and is driven outwards by fluid pressure, and blebs are being increasingly recognised as an alternative to pseudopods to drive cell motility, particularly when cells face mechanical resistance (Yoshida and Soldati, 2006; Zatulovskiy et al., 2014; Tyson et al., 2014). Blebbing induced by ATP differs in interesting ways from that induced by the chemoattractant cAMP (Langridge and Kay, 2006): in particular, it starts more quickly, there is no global polymerisation of actin, and neither PI3-kinase nor the MAP kinase, ErkB, are activated. Surprisingly, blebbing induced by ATP does not depend on TrpP, implying that another purinergic receptor must be responsible.

TrpP is homologous to the polycystin-2 or TRPP class of vertebrate Trp channels, which are also found in non-metazoan organisms (Venkatachalam and Montell, 2007) and include the human PKD2 protein (Mochizuki et al., 1996; Wu et al., 1998). PKD2 has been studied intensively as a cause of the severe genetic disorder autosomal dominant polycystic kidney disease, in which fluid-filled cysts grow within the kidney, and eventually disrupt its function (Chapin and Caplan, 2010). PKD2 cooperates with a large extracellular protein, PKD1, and together they can form plasma membrane cation channels of high calcium permeability (Hanaoka et al., 2000; Gonzalez-Perrett et al., 2001; Yu et al., 2009). However, we can detect no clear homologue of PKD1 in the *Dictyostelium* genome (Eichinger et al., 2005), and if one exists, it must be very divergent.

To our knowledge, no gating agonist has been reported for PKD2. In electrophysiological experiments it has been suggested to have an appreciable intrinsic conductance (Gonzalez-Perrett et al., 2001), although this is disputed (Yu et al., 2009), and there is also the possibility of mechanical gating. Our results showing that the primary response to extracellular ATP in *Dictyostelium* is mediated by a PKD2 homologue, is therefore both surprising and promising, raising the possibility that gating by ATP may be a more widespread feature of these channels.

## MATERIALS AND METHODS

### Cell cultivation, development, transfection and selection

Ax2 (Kay Laboratory strain; dictyBase DBS0235521), with minimal chromosomal duplications (Bloomfield et al., 2008) was used as parental stock; strains are listed in Table S5, and were renewed from frozen stocks every month. Cell procedures were at 22°C, unless otherwise stated. Cells were grown in HL5 with glucose (Formedium), plus 200 µg/ml dihydrostreptomycin, either in shaken suspension at 180 rpm, or in tissue culture dishes (Hirst et al., 2015). Development was initiated by washing cells free of growth medium in KK2C (16.5 mM KH_2_PO_4_, 3.9 mM K_2_HPO_4_, 2 mM MgSO_4_, 0.1 mM CaCl_2_ pH 6.1) and settling 1×10^8^ cells from 4 ml onto 30 ml of 1.8% Oxoid L28 agar/KK2C in a 9 cm diameter petri dish. After 10 min, excess buffer was aspirated off. Submerged development was observed with 2×10^6^ cells under 2 ml of KK2C in 3.5 cm tissue culture dishes.

Total RNA was extracted (RNeasy kit, Qiagen) from 5×10^7^-1×10^8^ developing cells and cDNA synthesised from 10.5 µg RNA for each timepoint (SuperScript First-Strand Synthesis System for RT-PCR, Life Technologies) using oligo(dT)_12-18_ for semi-quantitative PCR or a 1:1 mixture of random hexamers/oligo(dT)_12-18_ for cDNA cloning. Standard curves were established with cDNA dilutions (1:10 to 1:20,000) for each primer pair. The PCR reaction contained in 50 µl: 50 pmole of each primer, cDNA, 2 mM MgCl_2_, 200 µM dNTPs and 2.5 units Taq polymerase; PCR was run for 25 cycles.

Cells were transformed by electroporating 17.5 µg of gene disruption cassette, freed of plasmid backbone by restriction digest, or 30 µg of supercoiled plasmid DNA into 4×10^6^ cells (Pang et al., 1999; Hirst et al., 2015). Over-expression cell lines were selected and maintained in tissue culture dishes with HL5 plus 20-40 µg/ml G418, whereas *trpP* knockout clones were isolated by plating 60-240 cells/well in 96 well plates with 200 µl HL5 plus 10 µg/ml blasticidin S (InvivoGen). DNA was extracted from confluent wells after 10-14 days (Quick-gDNA MiniPrep, Zymo Research) and screened using primers PC2S26 and PC2S27 (primer sequences are given in Table S6) located outside the disruption cassette. Knockout clones were distinguished by the size of the PCR product and the presence of unique restriction sites introduced into the locus by the disruption cassette (Hirst et al., 2015).

### Plasmids

Primer sequences are given in Table S6. To construct the *trpP* knockout vector (pDT27) the 5′ homology was amplified using oligos PC2KO1 plus PC2KO2 and ligated into the *Apa*I site of pLPBLP (Faix et al., 2004) and the 3′ homology amplified using oligos PC2KO3 plus PC2KO4 and ligated as a *Not*I/*Sac*II fragment into the corresponding sites of the vector containing the 5′ homology. The *trpP* disruption cassette was liberated from pDT27 by digestion with *Kpn*I and *Sac*II prior to transfection of the cells. The *trpP* CDS (without a stop codon) was amplified by RT-PCR cDNA using oligos PCL5 plus PCL3 and ligated into the *Bam*HI/*Xho*I sites of pDT29 creating pDT33 with an in frame C-terminal fusion of GFP(S65T). The plasmid pDXA-3CΔ was made by digesting pDXA-3C with *Kpn*I and *Sac*I to remove the start codon from the A15 leader in the MCS (Manstein et al., 1995). GFP(S65T) was amplified using oligos RAGFP9 plus RAGFP10, then ligated into the *Xho*I site of pDXA-3CΔ giving pDT29. To construct *trpP* driven by its own promoter, a silent restriction site was introduced into pDT33 using mutagenic primers PC2S47 and PC2S48, changing +54A of the *trpP* CDS to +54T, giving a unique *Hind*III site. Primers PC2S48 and PC2S37 were used to amplify 940 bp upstream and part of the first exon of *trpP* from genomic DNA. The PCR product, digested with *Sal*I and *Hind*III, was ligated into the same sites within the mutated pDT33 thus exchanging the A15 for the *trpP* promoter, giving pDT42. Partial digestion of pDT42 with *Sal*I/*Xba*I removed the intact *trpP* CDS with its own promoter, which was ligated into *Xho*I/*Spe*I sites of pDM304 (Veltman et al., 2009) giving pDT41. The cameleons YC2.60 and YC3.60 (with *Aequorea victoria* codons) were removed from their pBIG vectors by partial digestion with *Bam*HI/*Sac*I and ligated into the same sites in pET28a (Horikawa et al., 2010), providing a template to amplify both cameleons using oligos YC367 plus YC368. The PCR products were ligated into the *Bam*HI/*Spe*I sites of the shuttle vector pDM344 (Veltman et al., 2009), removed as NgoMIV fragments and ligated into the corresponding site in pDT41 giving pDT48 (YC3.60) and pDT50 (YC2.60). Finally, pDT48 was used as the template to amplify the *trpP* CDS plus promotor using oligos PC2S69 plus PC2S70, with the product ligated into the *Xho*I/*Bgl*II sites of pDM323 (Veltman et al., 2009) giving pDT68.

### Microscopy

Vegetative cells were harvested from tissue culture plates, washed three times in HKC buffer (10 mM HEPES, 10 mM KCl, 250 µm CaCl_2_, pH 6.8) by centrifugation (300×***g*** for 2 min) and resuspended at 10^6^/ml in HKC. Cells were plated at 10^5^ cells/cm^2^ in 8-well Lab-Tek™ chambered coverslips with 300 µl HKC/well. Coverslips were incubated in a moist atmosphere for up to 1 h before use. Aggregation-competent cells were prepared by pulsing with cAMP for 3.5-5.5 h after 1 h starvation in shaking suspension (Traynor and Kay, 2007), or by plating 10^6^ washed cells per 35 mm tissue culture dish in 2 ml of HKC, incubating at 22°C for 1 h, then 15°C overnight (15-17 h), before returning to 22°C for at least 1 h, until they become elongated. Cells were harvested in fresh MKC by pipetting up and down and transferred to a chambered coverslip, where they normally formed long streams and aggregated after 2-4 h at 22°C. Confocal images were obtained using a Zeiss LSM 710 or 780 microscope with Zen 2010 software. Dunn chamber and micropipette chemotaxis assays and under-agarose motility assays were as described (Fets et al., 2014; Zatulovskiy et al., 2014). Images were analysed with ImageJ, Fiji (Schindelin et al., 2012) and Excel (Microsoft) software.

### Calcium imaging

Vegetative or aggregation-competent cells were challenged with effectors added as a 100 µl bolus at 4× final concentration to individual wells of a chambered coverslip. Mixing time was less than a second. Time-lapse images were collected on a Zeiss Axiovert 200 inverted microscope using a 40× C-Apochromat W Corr M27 lens (NA 1.2) and a Cascade II 512 EMCCD camera (Photometrics) controlled by Metamorph software (Molecular Devices). Cells were illuminated with a Lambda LS light source (Sutter Instruments) containing a 175W xenon bulb through a neutral density filter (Chroma Technology Corp, ND 2.0 A) with 1% transmittance and an excitation filter (ET436/20×, Chroma Technology Corp) that ensured only CFP was illuminated. The CFP and YFP emission light was separated using a beam splitter (Optical Insights, Dual View, filters D480/30, D535/40 and 505dexr dichromatic mirror). Exposure times were 100-250 msec and images captured at 3-7 per sec with a binning of 2 unless otherwise stated. Images were analysed using Jmalyse (Kerr and Schafer, 2006), Volocity (Perkin Elmer) and ImageJ. For each image the CFP (C) and YFP (Y) average fluorescence intensities, obtained by subtracting the background intensities (Y_bkg_ and C_bkg_) from the measured intensities (Y_meas_ and C_meas_) and ratios (R) were corrected for bleed-through from the CFP channel into the YFP (measured at 0.683) using Eqn (1) in the Microsoft Excel software package.
(1)
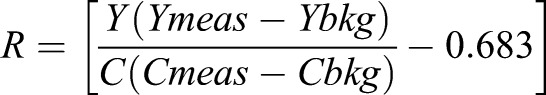
Response kinetics and peak areas were determined using GraphPad Prism.

### Rheotaxis

Sheer stress was created by a flow of KK2C driven by hydrostatic pressure in an Ibidi µ-Slide I ^0.2^ flow chamber (tissue culture treated, luer): 6.4 and 8.7 ml/min produced pressures of 3 and 4.5 Pa according to the manufacturer's lookup table (http://ibidi.com/fileadmin/support/application_notes/AN11_Shear). Cells in the chamber were filmed (1 frame/sec; binning of 2) using a Zeiss Axiovert S100 inverted microscope with motorised stage (Prior) and an ORCA-ER camera (Hammamatsu) controlled by µManager (Edelstein et al., 2014) software. ImageJ was used to analyse the movies.
